# High expression of carbonic anhydrase IX is significantly associated with glandular lesions in gastroesophageal junction and with tumorigenesis markers BMI1, MCM4 and MCM7

**DOI:** 10.1186/s12876-015-0310-6

**Published:** 2015-07-09

**Authors:** Aaron R. Huber, Dongfeng Tan, Jun Sun, David Dean, Tongtong Wu, Zhongren Zhou

**Affiliations:** 1 Department of Pathology and Laboratory Medicine, University of Rochester Medical Center, 601 Elmwood Avenue, Box 626, Rochester, NY 14642 USA; 2 Department of Biostatistics and Computational Biology, University of Rochester, Rochester, NY USA; 3 Department of Pediatrics, University of Rochester, Rochester, NY USA; 4 Department of Pathology, MD Anderson Cancer Institute, Houston, TX USA; 5 Biochemistry, Rush University Medical Center, Chicago, IL USA

**Keywords:** CA9, Esophageal adenocarcinoma, Esophageal squamous cell carcinoma, Barrett’s esophagus, Gastroesophageal reflux disease, BMI1

## Abstract

**Background:**

Carbonic anhydrase IX (CA9) is a transmembrane glycoprotein related to hypoxia. Increased CA9 expression has been associated with decreased survival and cancer progression and has been targeted as a potential therapy for several cancers, including esophageal cancer. The reported percentages of expression of CA9 in esophageal adenocarcinoma vary, and CA9 expression in precancerous esophageal lesions has not been well studied.

**Methods:**

In this study, we investigated CA9 expression in esophageal cancers and in precancerous lesions and explored the association of CA9 expression with prognostic factors and with stem cell and tumorigenesis-related markers including BMI1, cyclin E, ki67, MCM4 and MCM7 expression. Previously constructed tissue microarrays consisting of samples of 7 tissue types (columnar cell metaplasia, Barrett esophagus, low- and high-grade dysplasia, esophageal adenocarcinoma, squamous epithelium, and squamous cell carcinoma) were used for the immunostaining of CA9, BMI1, cyclin E, Ki67, MCM4 and MCM7.

**Results and discussion:**

CA9 high expression occurred more frequently in glandular mucosa with or without dysplasia than in squamous epithelium or squamous cell carcinoma. Survival duration of esophageal adenocarcinoma did not significantly differ between patients with high CA9 expression and those with low expression. High CA9 expression is significantly associated with BMI1, cyclin E, Ki67, MCM4 and MCM7 expression.

**Conclusions:**

High CA9 expression may be related to the acidic environment caused by gastroesophageal reflux disease in the gastroesophageal junction and associated with tumorigenesis through BMI1, MCM4 and MCM7.

## Background

The incidence of esophageal adenocarcinoma in the United States has increased dramatically, by over 600 %, in the past few decades [1–3]. Esophageal adenocarcinoma is an aggressive disease that has a poor prognosis, with an overall 5-year survival rate of approximately 17 % in the United States [2]. Barrett esophagus is characterized by metaplasia when goblet cells replace the normal squamous epithelium of the esophagus; it is a precancerous lesion that can lead to esophageal adenocarcinoma [1, 2]. Barrett esophagus and esophageal adenocarcinoma are both more common in men than in women [1, 2]. The association of Barrett esophagus with gastroesophageal reflux disease and with the development of glandular dysplasia and esophageal adenocarcinoma has been firmly established [1–3].

Carbonic anhydrase 9 (CA9) is a transmembrane metalloenzyme that enzymatically catalyzes the reversible hydration of carbon dioxide to bicarbonate and protons [4–8]. CA9 expression is often upregulated in cancer cells in response to hypoxic-acidic environments [4–8]. CA9 expression has been associated with tumor acid–base homeostasis under conditions of hypoxia, cancer progression, metastasis, and impaired response to traditional therapy [4, 5, 9] and is found in many types of cancer, including those of the kidney, bile duct, stomach, esophagus, lung, breast, cervix, ovaries, bladder, brain, head and neck, and oral cavity [4–15]. High expression of CA9 has been associated with poor prognosis and poor response to conventional chemoradiation therapy [4–15]. Inhibition of CA9 has been shown to slow tumor growth, inhibit metastasis, and decrease tumor stem cells in mice [16]. A few studies reported that CA9 was highly expressed in esophageal adenocarcinoma and esophageal squamous cell carcinoma [12, 14, 15, 17]. However, the percentages of expression of CA9 in these studies vary, and CA9 expression in precancerous lesions has not been well studied.

CA9 expression was reported to be associated with HIF-1α, Notch3, VHL and multiple genes in renal cell carcinoma and/or breast carcinoma [18–20]. Mutations of the common prolyl hydroxylation and pVHL binding domain lead to the loss of CA9 mRNA, and protein [19]. The *BMI1* gene is a stem cell marker that regulates proliferation [21] and has been observed in many solid tumors [22–24]. High expression of BMI1 has also been found in esophageal adenocarcinoma and esophageal squamous cell carcinoma [25–27]. Since CA9 was recently reported to play an important role in breast cancer stem cell survival [28], we investigated the association of BMI1 with CA9 in esophageal cancer. Cyclin E plays an important role in promoting the G1- to S-phase transition and is involved in several oncogenic functions [29, 30]. Amplification and overexpression of cyclin E have been reported in various cancers [31–33]. Recently, we found that the overexpression and amplification of cyclin E significantly increased comparing nondysplastic esophageal lesions to dysplastic lesions [34]. Since CA9 has been reported to functionally mediate tumor growth and metastasis [35], we investigated the relationship of CA9 and cyclin E expression in esophageal cancer.

Minichromosome maintenance (MCM) protein family consists of six related proteins that originally identified as having essential roles in initiation of DNA replication [36, 37]. The deregulation of the MCM proteins has been contributed to tumorigenesis and cell proliferation [38] and may be pre-cancer markers [39]. The relationship of MCM proteins with CA9 is unknown in esophageal lesions.

In current study, we first explored the relationship of CA9 high expression with gastroesophageal reflux disease including columnar cell metaplasia, Barrett’s esophagus, low-and high-grade dysplasia. Second, we examined the association of CA9 high expression with the clinicopathologic characteristics of esophageal squamous cell carcinoma and esophageal adenocarcinoma. Finally, we analyzed the association of CA9 overexpression with BMI1, cyclin E, MCM4, MCM7 and Ki67 expression in esophageal adenocarcinoma.

## Materials and methods

### Tissue microarrays for current study

The tissue microarrays were constructed with samples from 62 patients with squamous epithelium, 58 patients with columnar cell metaplasia, 27 patients with Barrett esophagus, 20 patients with low-grade dysplasia, 17 patients with high-grade dysplasia, 112 patients with esophageal adenocarcinoma and 23 patients with squamous cell carcinoma. Samples were collected from representative areas of formalin-fixed specimens. Three cores of each case were collected for the tissue microarray. All specimens were collected between 1997 and 2005, and the microarrays were constructed in the Department of Pathology and Laboratory Medicine at The University of Rochester Medical Center. We obtained clinicopathologic data, including patient age and sex, lymph node status, TNM stage, differentiation, and survival duration from the medical records. All patients’ identification was removed. This study is approved by The University of Rochester Medical Center RSRB office.

For this study, 5 micron sections were cut from the tissue microarrays and were stained with hematoxylin and eosin to confirm the tissue histology of each tissue core. Additional multiple sections were cut for immunohistochemical analysis. Four tissue cores were dislodged from the slides during the processing and were excluded from our study.

All 112 patients (99 men [88 %] and 13 women [12 %]) with esophageal adenocarcinoma samples included in the tissue microarray were treated with esophagectomy at the Strong Memorial Hospital (University of Rochester Medical Center; Rochester, NY) between 1997 and 2005. Patient age ranged from 34 to 85 years, with a mean of 65 years. The follow-up period after esophagectomy ranged from 0.3 to 142 months, with a mean of 39 months. All other patient specimens were collected at University of Rochester.

### Immunohistochemical analysis

Immunohistochemical analysis was carried out according to methods described previously, with some modifications [34, 40]. Briefly, tissue sections from the tissue microarrays were deparaffinized, rehydrated with graded alcohol, and washed with phosphate-buffered saline solution. Antigen retrieval for CA9, BMI1, cyclin E, MCM4, MCM7 and Ki67 was performed by boiling tissue sections in citrate buffer (pH 6.0; 10 mM) for 15 min. The tissues were permeabilized with 0.3 % Triton X-100 for 1 h at room temperature. After endogenous peroxidase activity was quenched and nonspecific binding was blocked, the sections were incubated with mouse monoclonal anti-CA9 (1:200; Thermo Fisher Scientific Pierce, Rockford, IL), anti-BMI1 (1:100; EMD Millipore, Billerica, MA), anti-cyclin E (1:100; Santa Cruz Biotechnology, Santa Cruz, CA), anti-MCM4 (1:50; Santa Cruz, CA), anti-MCM7 (1:50; Santa Cruz, CA) at 4 °C overnight, and anti-Ki67 (1:100; Santa Cruz, CA) at room temperature for 20 min. The secondary antibody (Flex HRP; Dako, Carpinteria, CA) was allowed to incubate for 30 min. After washing, sections were incubated with Flex DAB Chromogen (Dako, Carpinteria, CA) for 10 min and counterstained with Flex Hematoxylin for 5 min. Negative controls were obtained by replacing antibodies with normal serum.

**Fig. 1:**
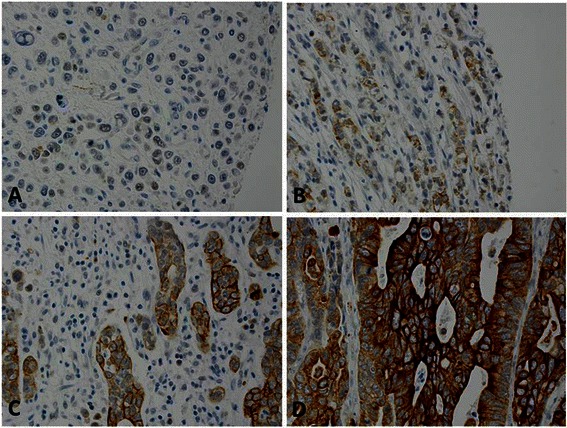
Intensity of carbonic anhydrase IX (CA9) expression in esophageal adenocarcinoma with immunohistochemical staining. Samples were scored from 0 to 3: **a**, Negative (0, no stain or weak stain in <10 % cells). **b**, Weakly positive (1, weak stain in ≥ 10 % cells). **c**, Moderately positive (2, moderate stain in ≥ 10 % cells). **d**, strongly positive (3, strong stain in ≥ 10 % cells). CA9 expression was predominantly distributed in the tumor cell membrane

All sections were reviewed independently by Z.Z. and A.R.H. Both pathologists were blinded to all clinical and pathologic information. Discrepancies were reviewed by both Z.Z. and A.R.H., and a final consensus was reached. The percentage (0 %–100 %) of cells that stained positive was determined. The intensity of CA9, BMI1 and cyclin E staining was graded as follows: 0, no stain or weak stain in < 10 % cells; 1, weak stain in ≥ 10 % tumor cells; 2, moderate stain in ≥ 10 % cells; or 3, strong stain in ≥ 10 % cells (Fig. 1). CA9, BMI1, and cyclin E protein expression was considered high if 10 % or more of cells stained with an intensity score of 2 or 3 (Fig. 1). The intensity of MCM4, MCM7, and Ki67 immunostaining was graded as 0 (negative) or 1+ (positive). The percentage of cells with positive nuclear immunostaining, ranging from 0 to 100 % was recorded in the increments of 10.

### Statistical analysis

Summary data are expressed as means ± standard deviations (SD) for continuous variables and as percentages for categorical variables. All statistical tests were 2-sided. *P* values less than 0.05 were considered statistically significant. Kaplan-Meier survival analysis and the log-rank test were used to analyze overall survival between the high CA9 expression group and the low CA9 expression group in esophageal adenocarcinoma. To assess the association of the clinicopathologic characteristics with CA9 expression, we used t-tests, Pearson chi-square tests, and Fisher exact tests as appropriate. A univariate logistic model with the histologic group as the sole explanatory variable of high CA9 expression was used, and contrast tests were used to compare the rates of high CA9 expression among the histologic groups. Statistical analyses were performed using SAS software, version 9.3 (SAS Institute, Inc., Cary, NC).

## Results

### High CA9 expression in esophageal adenocarcinoma and precancerous lesions

**Fig. 2:**
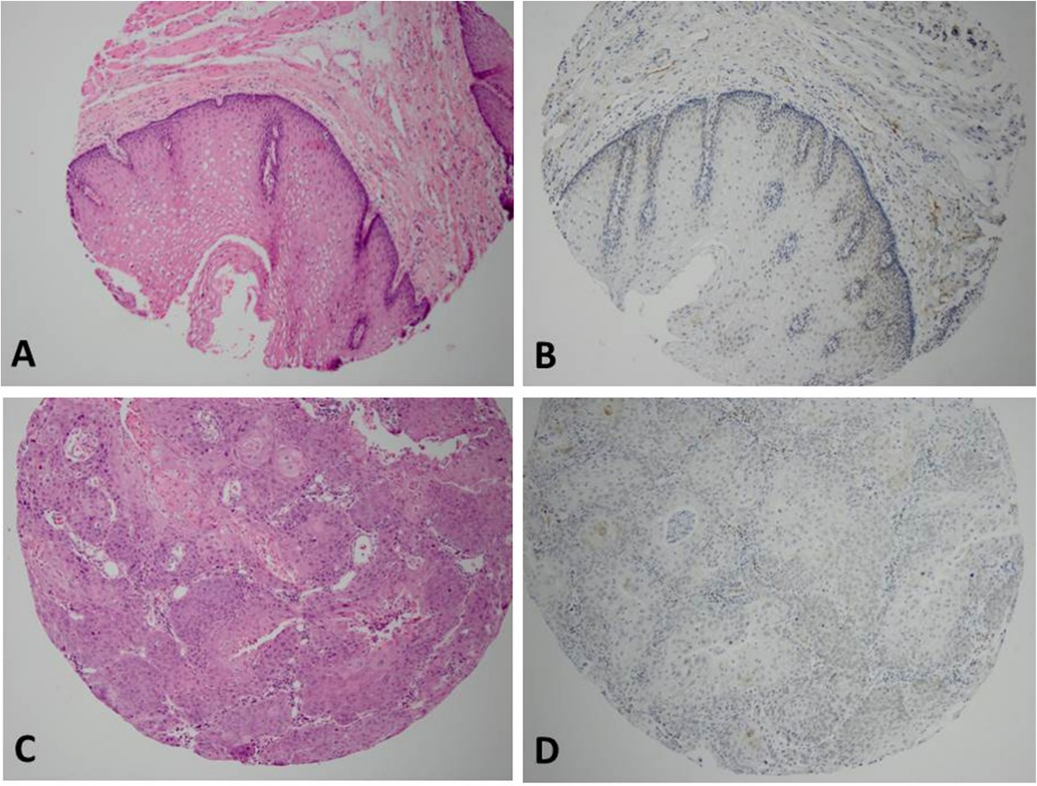
Squamous epithelium and esophageal squamous cell carcinoma samples showed no high intensity of carbonic anhydrase IX (CA9) expression. **a**. Squamous epithelium with H&E stain; **b**. No high CA9 expression in squamous epithelium; **c**. Squamous cell carcinoma with H&E stain; **d**. No high CA9 expression in sqaumous cell carcinoma

**Fig. 3:**
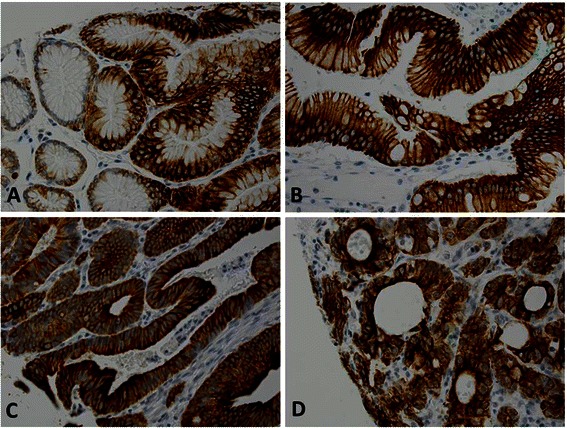
High carbonic anhydrase IX (CA9) expression in precancerous lesions. The samples showed strongly positive staining predominantly in the cell membrane. **a**, Columnar cell metaplasia (score 3). **b**, Barrett esophagus (score 3). **c**, Low-grade dysplasia (score 3) **d**, High-grade dysplasia (score 3)

Immunostaining results showed that CA9 was predominantly expressed in the cell membrane and rarely in the cytoplasm (Fig. 1). Of the tissue microarray cases, 90 % (101/112) esophageal adenocarcinoma, 94 % (16/17) high-grade dysplasia, 85 % (17/20) low-grade dysplasia, 74 % (20/27) Barrett esophagus, 83 % (48/58) columnar cell metaplasia, 9 % (2/23) squamous cell carcinoma, and 0 % (0/62) squamous epithelium had high CA9 expression (Table 1, Fig. 1, 2 and 3). The rate of high CA9 expression in patients with squamous cell carcinoma or squamous epithelium was significantly lower than that in patients with esophageal adenocarcinoma, high-grade dysplasia, low-grade dysplasia, Barrett esophagus, or columnar cell metaplasia (Table 2). High CA9 expression occurred predominantly in columnar cell lesions but was rare or absent in squamous cell carcinoma and squamous epithelium (Fig. 2).

**Table 1:** Rate of high carbonic anhydrase IX (CA9) expression in each histologic group

Histologic type	Total (n)	High expression	Low expression
		N (%)	N (%)
Adenocarcinoma	112	101 (90 %)	11 (10 %)
High-grade dysplasia	17	16 (94 %)	1 (6 %)
Low-grade dysplasia	20	17 (85 %)	3 (15 %)
Barrett esophagus	27	20 (74 %)	7 (26 %)
Columnar cell metaplasia	58	48 (83 %)	10 (17 %)
Squamous cell carcinoma	23	2 (9 %)	21 (91 %)
Squamous epithelium	62	0 (0 %)	62 (100 %)

**Table 2:** Comparison of high carbonic anhydrase IX (CA9) expression rates among histologic groups

Histologic comparison	*P* value
Adenocarcinoma vs.	
Barrett esophagus	.0312
Columnar cell metaplasia	.1683
High-grade dysplasia	.6066
Low-grade dysplasia	.4918
Squamous cell carcinoma	< .0001^**^
Squamous epithelium	< .0001^**^
Barrett esophagus vs.	
Columnar cell metaplasia	.3543
High-grade dysplasia	.1241
Low-grade dysplasia	.3706
Squamous cell carcinoma	.0002^**^
Squamous epithelium	< .0001^**^
Columnar cell metaplasia vs.	
High-grade dysplasia	.2684
Low-grade dysplasia	.8167
Squamous cell carcinoma	< .0001^**^
Squamous epithelium	< .0001^**^
High-grade dysplasia vs.	
Low-grade dysplasia	.3894
Squamous cell carcinoma	< .0001^**^
Squamous epithelium	< .0001^**^
Low-grade dysplasia vs.	
Squamous cell carcinoma	<.0001^**^
Squamous epithelium	<.0001^**^
Squamous cell carcinoma vs.	.4776
Squamous epithelium	.4776

^**^Indicates significance using the Bonferroni correction with α = 0.05/21 (0.00238)

### Association of high CA9 expression with clinicopathologic characteristics in esophageal cancer

We intended to analyze the association of high CA9 expression with clinicopathologic features in the esophageal adenocarcinoma and squamous cell carcinoma groups. Because only 1 patient with squamous cell carcinoma had high CA9 expression (1/23), we could not evaluate the association of CA9 expression with clinicopathologic characteristics for the squamous cell carcinoma group. None of the clinicopathologic characteristics were significantly associated with intensity of CA9 expression in the esophageal adenocarcinoma group (Table 3).

**Table 3:** Relationship of high carbonic anhydrase IX (CA9) expression and clinicopathologic characteristics in the esophageal adenocarcinoma group (*N* = 112)

Variable	High expression n (%)	Low expression n (%)	*P* value
Age, years			
Mean (SD)	64.8 (10.8)	63.5 (12.0)	.7260
Range	(34–85)	(36–80)	
Sex			
Male	89	10	.7598
Female	12	1	
Lymph node metastasis			
Positive nodes	3.7 (4.5)	3.9 (6.5)	.8752
T Stage			
0	1	0	.5618
1	10	2	
2	30	2	
3	60	7	
N stage			
0	27	2	.9260
1	49	6	
2	15	2	
3	10	1	
TNM stage			.3000
1	13	3	
2	22	1	
3	64	6	
Differentiation			
Poor	68	5	.3196
Moderate	32	4	
Well	5	1	

*TNM* Tumor Staging based on WHO Classification of Malignant Tumors; *N stage* based on lymph node metastasis

In esophageal adenocarcinoma and precancerous tissue groups, the patients with the high CA9 expression are 188 in male and 19 in female; the patients with the low or no CA9 expression are 89 in male and 29 in female. The overall association of high CA9 expression with male sex was significant (*P* = .0011). The odds of high CA9 expression were 2.8 times higher in men than in women.

### Overall survival analysis

We analyzed the effect of high CA9 expression on overall survival in patients with esophageal adenocarcinoma or squamous cell carcinoma. However, we could not obtain mean or median survival duration for patients with high CA9 expression in the squamous cell carcinoma group since only 1 of these patients showed high CA9 expression. The median survival duration of patients in this group with low CA9 expression was 26 months, with a mean survival of 37 months.

The median survival duration for patients with esophageal adenocarcinoma in the high CA9 expression group was 20 months, with a mean survival of 40 months. The patients with low CA9 expression had a median survival of 20 months, with a mean survival of 41 months. The survival duration did not significantly differ between patients with high CA9 expression and low CA9 expression (*P =* .8488).

### High CA9 expression is associated with multiple markers including BMI1, cyclin E, MCM4, MCM7 and Ki67

The rate of high CA9 expression was statistically associated with BMI1, cyclin E high expression and MCM4, MCM7 and Ki67 expression (*P <* .001) with Pearson correlation method. The correlation coefficient is positive related between CA9 expression with cyclin E (r = .1425), Ki67 (r = .1288), BMI1 (r = .2852), MCM4 (r = .2946) and MCM7 (r = .3106).

## Discussion

High CA9 expression was present in most glandular lesions but occurred rarely in squamous cell carcinoma; squamous epithelium did not show high CA9 expression. High CA9 expression maybe not significantly associated with the progression of precancerous esophageal disease to esophageal adenocarcinoma since the high CA9 expression did not show significant difference among all glandular lesions. However, high CA9 expression was significantly more frequent in glandular lesions than in squamous epithelium (0 %) and squamous cell carcinoma (9 %). CA9 could be a unique marker to differentiate adenocarcinoma and squamous cell carcinoma in poorly differentiated esophageal cancer.

The high CA9 expression in esophageal glandular lesions could be related to the hypoxic-acidic environment and columnar cell survival. Gastroesophageal reflux disease is a major risk factor for columnar cell metaplasia, Barrett esophagus, low- and high-grade dysplasia, and esophageal adenocarcinoma. Acid reflux from the stomach damages the cell junctions and causes dilation of the intercellular space; this acidic environment in the gastroesophageal junction and distal esophagus damages squamous epithelium and causes columnar cell metaplasia. CA9 is a well-known hypoxic indicator and plays an important role in maintaining the intracellular pH despite increased extracellular acidosis [17], thereby promoting cancer cell survival and growth in hypoxic-acidic environments. High levels of CA9 in columnar cell metaplasia and other precancerous glandular lesions may protect glandular cell survival in the acidic environment induced by gastroesophageal reflux disease. Therefore, most of the glandular tissues in our study showed high CA9 expression, with no significant difference between groups. In contrast, the very low frequency of high expression of CA9 in normal squamous epithelium and squamous cell carcinoma may be attributable to these tissues’ non-involvement by acid reflux.

The reported expression of CA9 in esophageal adenocarcinoma has varied from 47 to 80 % [12, 14, 17]. Conflicting results, ranging from 0 to 100 %, have also been published for CA9 expression in gastric adenocarcinoma [41–43]. In our study, the rate of high CA9 expression was very high in esophageal adenocarcinoma (90 %) and precancerous lesions (74 %–94 %) but was very low in esophageal squamous cell carcinoma (9 %). Several studies showed high CA9 expression in esophageal squamous cell carcinoma, ranging from 41 to 100 % [11, 14, 17]. Tanaka et al. found high expression of CA9 in 63 of 127 squamous cell carcinoma samples (50 %) [11]. However, there was a discrepancy in their results; the squamous cell carcinoma tissues seemed to have high CA9 expression, but this intensity of CA9 high expression in their figure was lower than that of the positive control (columnar cell metaplasia). Our findings show that patients with squamous cell carcinoma had low or no CA9 expression. The discrepancy between our findings and those of Tanaka et al. could be explained by the difference in our criteria for high expression. The antibodies and methods used in these esophageal cancer studies were also different. In addition, locations or races are different in all these studies. Therefore, the different percentages of CA9 high expression may be related with multiple factors including races, locations, antibodies and methods.

Several factors, such as the antibodies, the grading criteria, cancer heterogeneity, or immunostaining protocols, could explain these conflicting results about CA9 expression. We used the intensity of CA9 expression in glandular cells as the standard intensity. Our criterion for high CA9 expression was 10 % or more cells with an intensity score of 2 or 3. With our standard, the squamous cell carcinoma samples had low or no CA9 expression. Additionally, we used tissue microarrays for the immunohistochemical analysis: all tissue samples were automatically processed in the same conditions and at the same time in our pathologic laboratory. It is not possible that the lower intensity of squamous epithelium and squamous cell carcinoma was due to the immunohistological method variations.

Many studies have shown that CA9 high expression is associated with worse prognosis in solid tumors than lower or no CA9 expression in them [11, 12, 17]. However, in our study, high CA9 expression was not associated with worse prognosis, which may be due to the very high percentage of esophageal adenocarcinoma samples with high CA9 expression (90 %). Only a few cases (11/112; 10 %) did not show CA9 high expression. Additionally, the high CA9 expression was not associated with clinicopathologic features such as age, lymph node metastasis, tumor stage, and prognosis but was significantly associated with male sex (*P* = .0011). Men also have a higher risk for esophageal adenocarcinoma than women (men: women, 7–10:1). Our finding of the significant association of high CA9 expression with male sex implies that CA9 could be associated with men’s higher risk for esophageal adenocarcinoma.

Inhibition of CA9 with sulfonamide and/or coumarin inhibitors was recently shown to lead to a potent retardation for the growth of both primary tumors and metastases [44]. In addition, RENCAREX, a cG250 antibody (one of specific anti CA9 antibodies) underwent phase I-III clinical trials for treatment of patients with RCC. Phase I-II showed promising effects on metastatic renal cell carcinoma patients’ survival rate and tolerance of the antibody [45]. High CA9 expression in esophageal adenocarcinoma suggests that CA9 could be a therapeutic target in this cancer.

High CA9 expression was significantly associated with high BMI1 expression in esophageal adenocarcinoma and precancerous lesions. In our previous studies, the percentage and intensity of BMI1 expression significantly increased during the progression from columnar cell metaplasia to Barrett esophagus, low- and high-grade dysplasia, and adenocarcinoma [25]. We previously found that cells with high BMI1 expression had different distribution patterns in different types of tissue: mostly within the bottom of metaplastic columnar cell tissue compared with the full-gland distribution in dysplasia and adenocarcinoma. BMI1 is a stem cell marker that regulates self-renewal and differentiation; BMI1 also plays a role in cell-cycle regulation and carcinogenesis [46]. A recent study reported that CA9 plays an important role in the expression of epithelial-mesenchymal transition in breast cancer and is a critical mediator of the expansion of breast cancer stem cells [28]. CA9-specific small molecule inhibitors restricted the expansion of cancer stem cells. Our findings of the significant association of high CA9 expression with BMI1 imply that CA9 may be involved in esophageal tumorigenesis through esophageal stem cells.

High CA9 expression was significantly associated with cyclin E expression in esophageal adenocarcinoma and precancerous lesions. However, the correlation was low (r = .1425). Because CA9 maintains intracellular pH and helps tumor cells survive in hypoxic-acidic environments, CA9 may not be directly associated with cyclin E, which helps regulate the cell cycle.

High CA9 expression was significantly associated with MCM4, MCM7 and Ki67 expression in esophageal adenocarcinoma and precancerous lesions. However, the correlation was low (r = .1388) with Ki67 and relative higher (r = .2946; r = .3106) with MCM4 and MCM7. MCM4 and MCM7 are the part of MCM protein family, which play an essential roles in initiation of DNA replication [36, 37] and may be pre-cancer markers [39]. The deregulation of the MCM proteins has been contributed to tumorigenesis and cell proliferation [38]. The correlation of high CA9 expression with MCM4 and MCM7 in precancerous lesions implies that CA9 may play a role of tumorigenesis in the early stage. Ki67 is a traditional proliferation marker. The lower correlation of high CA9 expression with Ki67 indicates that CA9 may not be directly associated with cell proliferation.

## Conclusion

High CA9 expression may be related to the acidic environment in the gastroesophageal junction caused by gastroesophageal reflux disease. We found that high CA9 expression was associated with BMI1, MCM4 and MCM7, which indicate that CA9 may play a role in early tumorigenesis. CA9 was not a significant prognostic marker in patients with esophageal adenocarcinoma but may be associated with men’s high risk for esophageal adenocarcinoma. Its high expression in esophageal adenocarcinoma suggests that CA9 could be a therapeutic target in this cancer.

## Abbreviations

IHC: Immunohistochemistry
BE: Barrett’s esophagus
EAC: Esophageal adenocarcinoma
CM: Columnar cell metaplasia
LGD: Low grade dysplasia
HGD: High grade dysplasia
VDR: Vitamin D receptor
